# Accuracy of self-reported physical activity in patients with anorexia nervosa: links with clinical features

**DOI:** 10.1186/s40337-019-0258-y

**Published:** 2019-08-23

**Authors:** Louise Bezzina, Stephen Touyz, Sarah Young, Nasim Foroughi, Stacy Clemes, Caroline Meyer, Jon Arcelus, Sloane Madden, Evelyn Attia, Kathleen M. Pike, Phillipa Hay

**Affiliations:** 1 University of Sydney, Sydney, Australia; 2 School of Medicine, Western Sydney University, Sydney, Australia; 3 Loughborough University, Loughborough, UK; 4 University of Warwick, Coventry, UK; 5 University of Nottingham, Nottingham, UK; 6 Columbia University, New York, USA; 7 Translational Health Research Institute, School of Medicine, Western Sydney University, Locked Bag 1797, Penrith, South NSW 2715 Australia

**Keywords:** Anorexia nervosa, Physical activity, Self-report, Accelerometry, Compulsive exercise, Anxiety, Depression, Motivation to change

## Abstract

**Background:**

High levels of physical activity (PA) have long been described in patients with Anorexia Nervosa (AN). Despite the importance of measuring PA in this population, there are two important factors that remain unknown. First, it is not clear how accurate self-report measures of PA are among patients. Second, little is known about how clinical characteristics are associated with the accuracy of self-reported PA. Therefore, this study aimed to examine the accuracy of self-reported PA compared to an objective measure of PA in patients with AN. It also investigated whether levels of accuracy/inaccuracy were associated with compulsive exercise, motivation to change, and psychological distress.

**Method:**

Data were analysed from 34 adult outpatients with AN. Patients wore an accelerometer device (ActiGraph) for 4 days and completed a retrospective self-report measure of exercise (Exercise Participation Screening Questionnaire). They also completed measures of compulsive exercise (Compulsive Exercise Test), motivation to change (The Anorexia Nervosa Stages of Change Questionnaire), and psychological distress (Kessler-10).

**Results:**

On the self-report measure, patients accurately reported their time spent in moderate and vigorous intensity PA, however, they significantly under-reported their light physical activity (compared to the accelerometer data). Accurate reporting of total PA was positively associated with higher levels of compulsive exercise. There was evidence to suggest that clinical features, such as motivation to change and psychological distress, may be associated with inaccurate reporting at some levels of PA intensity and not others.

**Conclusions:**

Results indicate that patients with AN are likely to under-report their light intensity PA. We also found preliminary evidence for how compulsive exercise, motivation to change, and distress are associated with self-reported PA accuracy. Clinical implications and directions for future research are considered.

**Trial registration:**

ACTRN12610000585022. Taking a LEAP forward in the treatment of anorexia nervosa: a randomized controlled trial. NHMRC grant: 634922.

## Plain English summary

High levels of physical activity are common in people with anorexia nervosa and are associated with poorer physical and psychological outcomes. It is important to measure the amount of activity or exercise a person is engaging in as part of assessment and treatment. This study looked at whether a self-report questionnaire (answering a survey) would be accurate in measuring physical activity. We compared the patient’s self-reported physical activity to that estimated from an accelerometer (pedometer) device worn around the person’s waist. We found that people under-reported their light activity on the self-report questionnaire (e.g. walking), but did not under-report their moderate or vigorous activity. We then looked at whether the accuracy of self-reported activity was related to a tendency to be compulsive about exercise, motivation to recover from anorexia nervosa, and psychological distress. We found that people whose exercise was more compulsive had more accurate self-reported total exercise. There was weak evidence to suggest that motivation to change and psychological distress may be associated with inaccurate reporting at different levels of exercise intensity. The results may help us better understand the usefulness of self-report exercise measures, and when accelerometer devices may be more appropriate. Results may also help the development of more accurate self-report measures and treatment programs for people with anorexia nervosa.

## Background

Anorexia nervosa (AN) is a psychological illness characterised by low body weight, body image distortion and an intense fear of gaining weight [1]. Fear of weight gain often manifests through dietary restriction and often coincides with increased levels of physical activity (PA). High levels of PA have long been described in patients with AN [2], and studies have found that 31–80% of patients with AN engage in high levels of PA [3–5]. High levels of PA in patients with AN have been associated with further negative physical [6–8] and psychological [8, 9] implications.

There is increasing recognition that measuring an individual’s level of PA is an important aspect of assessment and treatment in patients with AN [10]. In clinical settings, PA is typically measured either using observation or self-report measures including: observation by clinical experts [11], questionnaires [12], semi-structured interviews [13], and activity diaries [14]. However, there has been concern that these measures may not accurately reflect the level of PA engaged in due to dependence on factors such as patients’ recall and accuracy of patient reporting [15]. The potential for bias in self-report measures has led to a suggested shift towards measuring PA using direct or objective measures that do not rely on the patient’s self-report [15, 16]. The most commonly used direct methods for studying PA in patients with AN has been accelerometry [15]. Accelerometer devices are typically attached to the wearer at the wrist or hip, and measure activity using acceleration signals. These signals can then be converted to units of energy expended, and / or summarised as time spent in different intensities of PA, using standardised cut points such as sedentary, light, moderate, and vigorous PA [17].

Research has explored the extent to which self-report measures reliably measure PA, compared to accelerometer data, across various populations and contexts [18]. Indeed, a recent systematic review found that non-clinical samples tended towards overestimating their PA on self-report measures compared to accelerometer devices [18]. Although this trend was apparent in both genders, it was particularly strong among females who self-reported 138% more PA than was recorded on accelerometer devices [18]. It has also been found that when stratified by intensity of activity, non-clinical samples are most accurate when reporting time in light intensity PA (such as slow walking) and least accurate when reporting higher intensity PA (such as brisk walking, jogging or running) [18].

There are mixed findings regarding the accuracy of self-report measures of PA in patients with AN. To date, four have compared self-report measures to objective measures of PA in patients with AN. One such study compared self-reported PA levels over three days on a visual analogue scale (0–10) to an accelerometer (Actiwatch) in 18 patients with AN [16]. It was found that patients under-reported their PA on self-report measures and there was no correlation between self-reported PA and accelerometer data. Similarly, a study by Bratland-Sanda [19] asked seven inpatients with AN to record the type, frequency, duration (minutes / session), and intensity (using Borg’s 6–20 rating of perceived exertion scale) of their PA. It was shown that patients significantly under-reported their moderate-vigorous physical activity on the self-report diary compared to an accelerometer (ActiGraph MTI model 7164; Manufacturing Technology, Fort Walton Beach, FL) over a seven-day period. There was no significant difference between self-report and objective measures in the nonclinical control group. Finally, Alberti et al. [20] demonstrated that 52 inpatients with AN significantly under-estimated PA on a self-administered format of the International Physical Activity Questionnaire (IPAQ) compared to an accelerometer device (Actiheart) over a seven-day period.

In contrast, Keyes and colleagues [21] found that patients with AN were more likely than controls to report higher PA on self-report measures compared to accelerometer data. In this study, self-reported PA on the IPAQ was compared to accelerometer data (Actiwatch AW4; Cambridge Neurotechnology, Cambridge, UK) in patients with AN [both inpatients (*n* = 18] and outpatients (*n* = 37)] and healthy controls over seven days. Accelerometer data indicated that patients with AN exercised for similar periods of time, and with a similar intensity, to healthy controls. However, patients with AN self-reported that they had engaged in more exercise than healthy controls. The authors hypothesised that in AN, self-report may overestimate PA due to differences in perception related to their eating disorder psychopathology [21]. Thus, while the research suggests that patients with AN are inaccurate in their self-reported PA compared to accelerometer data, evidence is conflicting as to whether they overestimate or underestimate their PA on self-report measures.

Limited research to date has investigated why patients with AN are inaccurate in their self-reported PA. One factor that may contribute to the accuracy of self-reported PA may be the nature of the PA itself. According to Adkins and Keel [22], dysfunctional exercise in eating disorders may have two dimensions. The first is the quantitative dimension, such that exercise is excessive as defined by its frequency, intensity and duration. The second is the qualitative dimension, or the compulsive nature of the exercise. Compulsive exercise is defined as a rigid and highly driven urge to exercise, and there is a perceived inability to cease despite the risk of harmful consequences [23]. Meyer and colleagues [24] developed a model of compulsive exercise in eating disorders, wherein compulsive exercise is maintained by factors including perfectionism, rigidity, eating disorder pathology, compulsivity, and psychological dependence. High levels of compulsive exercise have also been found to be positively associated with record keeping [25]. As such, it is important to understand whether patients with higher levels of compulsive exercise may be more accurate on self-report measures, as they may be more rigidly adhering to their goals, and therefore more aware, of their PA levels.

Another factor that might be related to the accuracy of self-reported PA is an individual’s motivation to change and recover from anorexia nervosa. For instance, in a study by Bratland-Sanda and colleagues [19] one participant reported “I am not physically active – I only go for walks”, while also reporting that she walked one hour daily. The authors hypothesised that under-reporting may be deliberate and occur due to fear of mandatory increase of energy intake/ restriction of PA in the hospital/treatment program. As such, patients may not report light exercise as they would under-report their exercise participation [19]. Similar findings were described in an interview study by Kolnes [26]. Specifically, patients under-reported and rephrased descriptions of their engagement with exercise, often implying that their activity (e.g. walking) would not necessarily be counted as exercise. Kolnes [26] reported that participants articulated a clear understanding of the need to reduce PA as part of their treatment program, but at the same time engaged in long periods of light activity. Thus, they suggested that a patient’s tendency to under-report their PA may be a way to convey adherence with treatment while still expending energy contributing to weight loss. It has been established that a key feature of AN is that it is ego-syntonic in nature, leading to poor insight and low motivation to change [27]. In general, lower motivation to change has been found to be associated with poorer patient outcomes in eating disorder treatment including lower BMI [28], slower weight gain [29], higher rates of compulsive exercise [8], and poorer quality of life [30]. The trans-theoretical model of motivation to change [31] has been used within the eating disorder field and suggests that patients may move between six stages of change. These stages include pre-contemplation, contemplation, preparation, action, maintenance and termination. As such, it may be expected that patients in the early stages of change may be more likely to under-report their PA. However, this hypothesis has not yet been tested.

Level of psychological distress may be another factor related to the accuracy of self-report measures of PA. Depression and anxiety symptoms are frequently comorbid with AN [32, 33]. The lifetime prevalence of major depressive disorder in patients with AN ranges from 65 to 81% [34], and the lifetime prevalence for an anxiety disorder is approximately 55% [35]. It is well established that depressed mood and anxiety symptoms are associated with a range of cognitive impairments including memory, inhibition, control, planning, attention, and flexibility [36–38]. It could thus be hypothesised that patients showing higher rates of psychological distress may also suffer cognitive impairments associated with inaccurate recall. These would be expected to impact the degree to which they could accurately report their PA on self-report measures.

In summary, there remains ambiguity in the literature regarding the accuracy of self-reported PA in patients with AN. It is not clear whether patients with AN will under or over-report their PA, and whether their accuracy is influenced by the intensity of activity. Furthermore, there is a paucity of research into the clinical features which may be associated with the accuracy of self-reported PA. Specifically, it has not yet been tested whether level of inaccurate reporting is associated with features such as level of compulsive exercise, motivation to change, and level of psychological distress. Therefore, this study has two aims.

### Aims

The first aim of the study was to explore the relationship between self-reported PA and accelerometer data in patients with AN. Given the previous research, it was expected that patients would under-report their PA, and may be more likely to under-report light PA such as walking.

The second aim of this study was to examine the clinical characteristics associated with inaccurate reporting of PA. Due to limited existing research in characteristics associated with under-reporting in AN, the following exploratory hypotheses were suggested. Firstly, it was hypothesised that there will be an association between higher levels of compulsive exercise and higher accuracy in their self-report. Secondly, that there would be an association between lower motivation to change and under-report their PA. Finally, that higher psychological distress may be associated with greater inaccuracy on self-report.

## Method

### Participants

This study was nested within a trial of “CompuLsive Exercise Activity TheraPy (LEAP): a new approach to compulsive exercise in anorexia nervosa” [39]. This study was a multi-site, randomised controlled trial (RCT) which aimed to test a novel cognitive behavioural therapy for outpatients with AN about what constitutes healthy exercise, and equip them with skills to participate in balanced exercise. Adults with AN (*n* = 78; 4 males) were recruited into the RCT. To be eligible for inclusion, participants had to be at least 18 years old, have a primary diagnosis of AN using the Eating Disorder Examination (EDE) [40] interview, have a Body Mass Index (BMI) between 14 and 18.5 kg/m^2^, and have exercised within the last month as indicated on the Exercise Participation Screening Questionnaire (EPSQ) or on the EDE-Q (at least one occasion in the past 28 days). Participants were recruited through public advertising, or referral from an eating disorder service.

All three recruitment sites approved ethics applications for the RCT: The Western Sydney University Human Research Ethics Committee in Australia; the National Health Service Research Ethics Committee in the UK, as part of the Health Research Authority; and the Institutional Review Board at Columbia University in New York, USA. Participants of the current accelerometer study (*n* = 36) were recruited only from Australia and the UK, as the US site did not collect accelerometer data. Written informed consent was provided by the participants for the treatment trial and completion of research measures. The Western Sydney University Human Research Ethics Committee (HREC) approved ethics for the current nested study and the addition of Louise Bezzina as an investigator (HREC approval number: H7732; amendment approved 11th September 2017).

At baseline, participants completed questionnaires and were asked to wear the accelerometer (GT3X+ ActiGraph, Manufacturing Technology, Fort Walton Beach, FL) on an elastic belt around their waist for 4 days (but not when in bed, while showering or swimming). The ActiGraph is an accelerometer device which has been validated in adults [41] and has been used in previous studies with patients with AN [19]. The software program ActiLife 6.9 was used for data processing and analysis. Sequences of more than 60 mins of continuous zero counts (indicating the accelerometer was not in use) were excluded from the analysis, and participants were required to have worn the device for at least 10 h during waking time for each day to be considered valid [42]. Freedson cut points [17] were used to convert the downloaded accelerometer counts per minute data into daily times (in minutes) participants spent in light, moderate, and vigorous intensity PA. Total minutes spent in PA per day were also calculated.

To meet inclusion for the current study, participants had to have worn the accelerometer for a minimum of 10 h per day for at least 2 days. Based on this criterion, 34 participants (32 female, 2 male) were included in the analysis of the current study. This comprised 24 participants from the Australian site, and 10 participants from the UK site. Analyses were completed on group differences between UK participants who wore the accelerometer and those who declined (50% of participants; at the UK site, accelerometer participation was optional), and no significant difference was found in eating disorder severity level as measured by the EDE global score. The average wear time was 14.4 h per day, for an average of 3.5 days. Demographic and clinical characteristics of the participants are found on Table 1.

**Table 1:** Demographic and clinical characteristics of the participants

Demographics / Clinical characteristics (*N* = 34)	M (SD)	Range (Min - Max)	95% CI
Age	26.3 (6.4)	18.3–29.1	24.1–28.5
Current BMI^a^	16.4 (1.2)	14.0–18.0	15.9–16.8
EDE^b^ Global Score	3.4 (1.2)	0.7–5.4	2.9–3.8
*“When do you think AN*^*c*^ *started?”* (age in years)	16.6 (4.8)	5–33	14.9–18.2
*“When was AN diagnosed?”* (age in years)	20.4 (6.6)	12–38	18.1–22.7
Years between diagnosis and enrolment in LEAP^d^	5.7 (6)	1.8–23.6	3.6–7.8
	N (%)		
Gender	Female: 32 (94.1%) Male: 2 (5.9%)		
AN Subtype (*n* = 32)	▪ AN-Restrictive subtype: 19 (59.4%) ▪ AN-Binge/Purge subtype^e^: 13 (40.6%) - Regular objective bulimic episodes: 7 (21.9%) - Self-induced vomiting: 3 (9.4%) - Laxative/diuretic use: 9 (28.1%)		
Country of birth	Australia: 21 (61.8%) United Kingdom: 9 (26.5%) Other: 4 (11.8%)		

^a^Body Mass Index, ^b^Eating Disorder Examination, ^c^Anorexia Nervosa, ^d^ CompuLsive Exercise Activity TheraPy (LEAP), ^e^ At least one behaviour, 4 or more episodes per month, over last 3 months

### Measures

Participants wore the accelerometer after their initial assessment with the research assistant. They also completed the following self-report questionnaires at baseline:

*The Exercise Participation Screening Questionnaire (EPSQ)* is a validated measure assessing common exercise types, with duration, frequency of sessions and intensity [43]. The EPSQ in its original form was administered as a respondent based interview over multiple time periods and we adapted this to a self-report format of current exercise. Thus, in this study it asked about exercise completed in the past four weeks, including the type of exercise, number of sessions per week, average duration per session, and self-rated intensity of each exercise. The types of exercise listed were walking, jogging, swimming, aerobics, weights, cycling, swimming, tennis and other. Participants frequently indicated pilates, yoga, resistance exercises (e.g., sit-ups, push-ups, lunges), and cardio machines as other. Participants self-rated the activity’s intensity level (low, medium, or high) and the duration of each activity in a typical session (in minutes). These intensity levels were used to compare to the accelerometer activity counts of light, moderate and vigorous respectively. A daily average of minutes/day was calculated for each level of intensity of exercise.

*The Eating Disorder Examination* (EDE) [40] is a semi-structured interview administered by the research assistant to assess the psychopathology associated with the diagnosis of an eating disorder. It is comprised of four subscales (eating concern, weight concern, shape concern, and dietary restraint), one global score, and ratings of eating disorder behaviours to assess severity of symptoms. The EDE has been demonstrated to have robust psychometric properties [44]. The Cronbach’s α was .89 in the current study.

*The Anorexia Nervosa Stages of Change Questionnaire (ANSOCQ)* [45] is a measure of motivation to change which has been validated in adult patients with AN. It consists of 20 questions, assessing three factors: weight change including motivation to reach a minimum healthy weight (weight gain), motivation to alter the relative importance of shape and weight compared to other aspects of life including accomplishments and fulfilment (eating, shape, and weight concerns), and motivation to improve relational and emotional concerns associated with AN (ego-alien aspects). Each question has 5 responses representing the various stages of change (pre-contemplation; contemplation; preparation; action; and maintenance) and the participant can select more than one statement per question, according to the answer/s which best represent their current beliefs. Example items include: *“There is no way I would be prepared to gain weight on these body parts”* (weight gain: pre-contemplation response)*; “I have decided that I need to do something about the fear I have of becoming fat because it is controlling me”* (eating, shape and weight concerns: contemplation response)*;* and *“My emotional problems have improved and I am trying to keep it this way”* (ego-alien aspects: maintenance response). Higher scores represent more motivation to change. A mean stage of change score is calculated: < 1.5 = pre-contemplation; 1.5–2.4 = contemplation; 2.5–3.4 = preparation; 3.5–4.4 = action; > 4.5 = maintenance. In the current study, the total score was used, and it’s Cronbach’s α was .91.

*Kessler-10 item distress scale (K*^*− 10*^*)* [46] is a 10-item measure of psychological distress including both anxiety and depression symptoms. The questionnaire includes questions such as *“Over the past four weeks (28 days), how often have you felt hopeless?”*. Higher scores indicate greater psychological distress, and the maximum score was 50. It has been shown to be valid and reliable in eating disorders research [47]. In the current study, the total score was used, and Cronbach’s α was .89.

*The Compulsive Exercise Test (CET)* [23] is a 24-item measure designed to assess the core features of compulsive exercise in eating disorders. It has 5 subscales: Avoidance and rule driven behaviour, reflecting exercise governed by rules and consequences (e.g., *“If I cannot exercise I feel low or depressed”);* Weight control exercise, reflecting exercise focused on shape and weight (e.g., *“I exercise to improve my appearance”);* Mood improvement, regarding the positively reinforcing effects of exercise on mood (e.g., “*I feel less anxious after I exercise*”); Lack of exercise enjoyment, meaning performing exercise in an obligatory way, despite not gaining enjoyment from it (e.g., “*I find exercise a chore*”); and Exercise rigidity, assessing the pattern of inflexibility associated with exercise routines (e.g., “*My weekly pattern of exercise is repetitive*”). The CET uses a 6-point Likert scale from 0 (never true) to 5 (always true). Means for the five subscales are summed for the CET-Total, with higher scores indicating a greater level of compulsive exercise. The CET has demonstrated strong reliability, validity, and clinical utility in adult patients with AN [12]. The total score was used in the current study, and it’s Cronbach’s α was .93.

### Statistical analysis

To address the first aim (i.e., explore the relationship between self-reported PA and accelerometer data in patients with AN), the discrepancy between self-reported PA and accelerometer data was calculated by subtracting the average time spent in PA based on accelerometer data from the self-reported PA (i.e. EPSQ - accelerometer = discrepancy). Negative values would therefore indicate under-reporting on the self-report measure (EPSQ). A paired-samples *t*-test (within-subjects) was then conducted to compare the total PA (averaged across intensity level) reported on the EPSQ and recorded on the accelerometer device. This analysis was then repeated as a paired-samples *t*-test when PA was stratified by level of intensity (i.e. light, moderate and vigorous).

To address the second aim (i.e. examine the clinical characteristics associated with inaccurate reporting of PA), correlational analyses were used to examine the association between inaccurate reporting and clinical variables. The Shapiro-Wilk test was first used to test the distribution of the data due to the small sample size. Some measures (e.g., CET, and the discrepancy between overall self-report of exercise and accelerometer data) were non-normally distributed. As all analyses involved correlations with at least one non-normally distributed variable, non-parametric tests (Spearman’s rho) were used in analyses. Based on the hypotheses, 2-tailed tests were used and correlational analyses were all within subjects. Results were considered significant when *p* <  0.05 and all statistical analyses were carried out using SPSS version 21.0.

## Results

### Accuracy of self-report (EPSQ) vs accelerometer data

The first aim of the study was to examine whether patients with AN accurately reported their total PA on the EPSQ compared to accelerometer data.

Results demonstrated that participants significantly under-reported their total minutes per day of PA on the EPSQ (*M* = 91.8, *SD* = 96.0) compared to accelerometer data (*M* = 325.6, *SD* = 109.1); *t* (33) = − 13.2, *p* <  0.001. This indicated that when averaged across level of intensity, patients were significantly under-reporting their PA on the EPSQ compared to accelerometer data. We investigated factors which may have been associated with accuracy of reporting. There was no significant difference in accuracy of reporting between restricting and binge/purge subtype *t* (30) = − 1.1, *p* = 0.3. Overall total PA accuracy was not correlated with eating disorder symptomatology (EDE global score), *r* (32) = .21, *p* = 0.23.

**Fig. 1:**
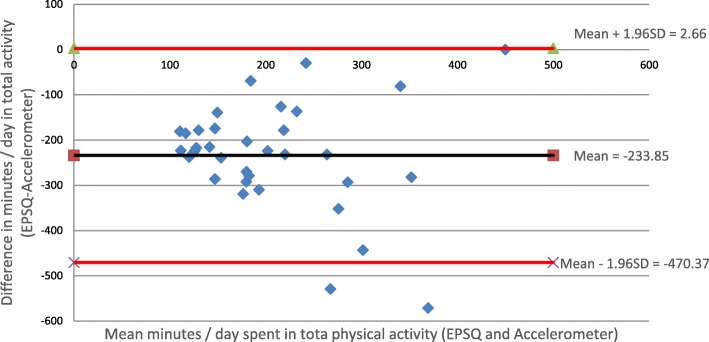
Bland-Altman plot showing difference versus average values of self-reported (EPSQ) and objectively measured (Accelerometer) total physical activity. The plots show discrepancy with 95% limits of agreement in patients (*n* = 34). Negative values indicate under-reporting of PA on the self-report measure (EPSQ)

A Bland-Altman Plot (Fig. 1) was used to further explore the level of agreement between self-report and accelerometer measures by deriving the discrepancy in total PA and the 95% limits of agreement [48]. The discrepancy was − 233.9 min / day and the limits of agreement were 2.7 and − 470.4 min / day. This large difference indicates a poor agreement between the EPSQ and accelerometer data in measuring total daily PA.

The discrepancy between EPSQ and accelerometer data was then calculated for light, moderate, and vigorous PA to determine whether patients were differentially under-reporting for different intensity levels of PA. The mean minutes per day in each level of intensity of PA (as recorded by the accelerometer and EPSQ) are summarised in Table 2.

**Table 2:** Mean minutes per day of physical activity recorded on accelerometers and EPSQ, and the discrepancy between these means

PA intensity	Accelerometer (mins/day)	EPSQ^a^ (mins/day)	Discrepancy	*p*
Light	258.9	17.8	− 241.1	< 0.001
Moderate	55.6	53.7	−1.9	0.80
Vigorous	11.2	20.3	9.1	0.28
Total	325.6	91.8	−233.9	< 0.001

^a^Exercise Participation Screening Questionnaire

When stratified by intensity of exercise, it appeared that the discrepancy for total PA could be related to under-reporting of light PA. A paired-samples *t*-test was conducted to compare light PA recorded on the EPSQ and accelerometer device. There was a significant difference in reporting light PA on the EPSQ (*M* = 17.8, *SD* = 53.0) compared to the accelerometer (*M* = 258.9, *SD* = 88.6); *t* (33) = − 11.3, *p* <  0.001. Results showed that participants were not significantly under reporting their moderate or vigorous PA (*p* > 0.05).

These results indicate that the under-reporting of light PA on the EPSQ explained the significant under-reporting of total PA.

### Links between inaccurate reporting and clinical features

Spearman’s correlational analyses were used to examine the relationship between under-reporting of exercise (total PA) and compulsive exercise, motivation to change, and psychological distress. The results of the correlations are summarised in Table 3.

**Table 3:** Spearman’s correlations between under-reporting on EPSQ (self-report vs accelerometer discrepancy) and compulsive exercise, motivation to change, and distress

Level of exercise discrepancy (EPSQ^a^ vs Accelerometer)	Compulsive exercise (CET^b^ total)	Motivation to change (ANSOCQ^c^ total)	Distress (Kessler-10)
Light	0.03	0.05	0.36* (*p* = 0.04)
Moderate	0.24	−0.39* (*p* = 0.02)	−0.01
Vigorous	0.12	−0.03	0.08
Total	0.36* (*p* = 0.04)	−0.06	0.29

^a^ Exercise Participation Screening Questionnaire, ^b^ Compulsive Exercise Test, ^c^ Anorexia Nervosa Stages of Change Questionnaire; *p<.05

**Fig. 2:**
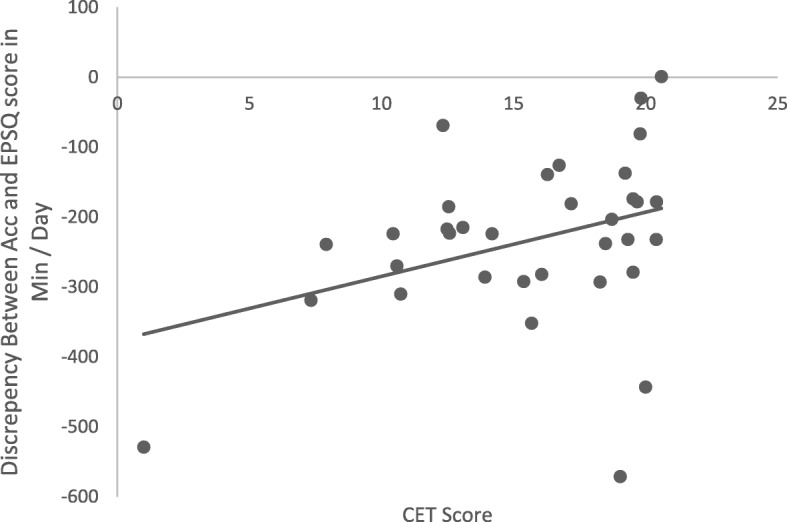
Correlation between the discrepancy of accelerometer data and EPSQ score, and compulsive exercise (CET score) (*n* = 34). Negative values indicate under-reporting of PA on the self-report measure (EPSQ)

The mean total compulsive exercise score was 15.6 (*SD* = 4.6). More accurate reporting of total PA on the EPSQ compared to accelerometer data was weakly, however significantly correlated with compulsive exercise, *r* (32) = .36, *p* = 0.04. This is represented in Fig. 2. This suggested that a higher level of compulsive exercise was associated with increased accuracy on self-report measures compared to accelerometer data. When PA was stratified by level of intensity, there were no significant correlations found between under reporting light, moderate, or vigorous PA and compulsive exercise.

**Fig. 3:**
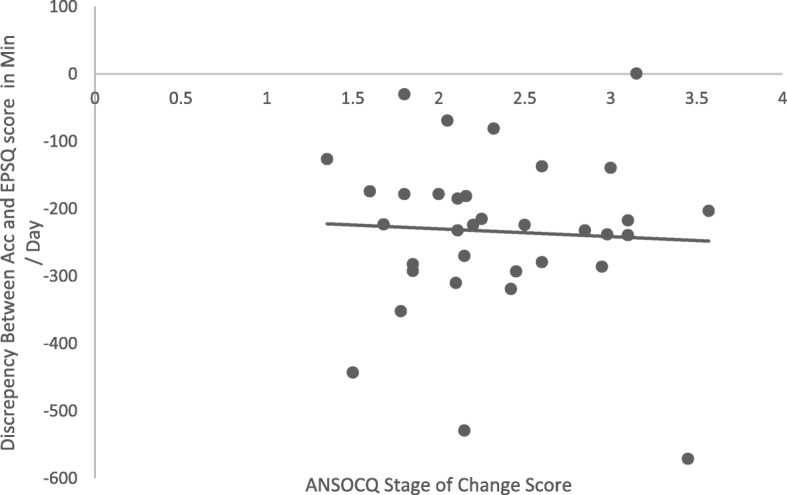
Correlation between the discrepancy between accelerometer rand EPSQ score and motivation to change (ANSOCQ score) (*n* = 34). Negative values indicate under-reporting of PA on the self-report measure (EPSQ)

The mean total score for stage of change was 46.4 (*SD* = 11.6). Under-reporting of total PA on the EPSQ compared to accelerometer data was not significantly correlated with stage of change (*p* > 0.05), as shown in Fig. 3. However, when PA was stratified by level of intensity, there was a significant correlation between under-reporting of moderate PA and motivation to change, such that those who were more motivated to change were more likely to under-report their PA, *r* (32) = −.39, *p* = 0.02.

**Fig. 4:**
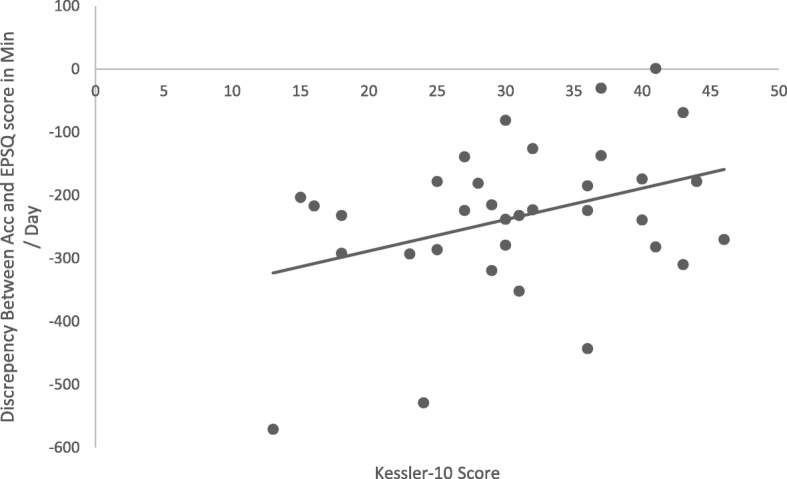
Correlation between the discrepancy between accelerometer rand EPSQ score and psychological distress (Kessler-10 score) (*n* = 34). Negative values indicate under-reporting of PA on the self-report measure (EPSQ)

The mean score for psychological distress was 31.0 (*SD* = 8.8). Under-reporting of total PA on the EPSQ compared to accelerometer data was not significantly correlated with psychological distress (*p* > 0.05), as shown in Fig. 4. However, when PA was stratified by level of intensity, this trend appeared to be driven by the significant relationship between the discrepancy of self-reported light PA and distress, *r* (32) = .36, *p* = 0.04.

## Discussion

The current study aimed to examine how accurately patients with AN self-reported their PA on the EPSQ compared to accelerometer data. Results indicated that patients with AN significantly under-reported their overall PA on the EPSQ compared to accelerometer data. It is difficult to compare these results to previous research outcomes given the different methods of self-reported and accelerometer PA assessment, length of time PA was monitored, and sample size. However, results were consistent with three previous studies which compared self-report measures to accelerometer data [16, 19, 20]. In contrast, this finding is inconsistent with the results of Keyes et al. [21], which found that patients with AN showed higher self-reported PA compared to healthy controls, while accelerometer-measured PA levels for patients with AN and controls were not significantly different. One possible explanation for this difference could be that their study did not include comparisons between the self-report and accelerometer data for each individual [21]. As such, individual differences in the accuracy of reporting may not have been captured. Further, several patients who had missing data on accelerometer devices were excluded from analyses, potentially underestimating accelerometer PA results.

A novel finding from the current study was that patients were more likely to under report their light PA compared to moderate and vigorous PA. Previous studies have not examined differential under-reporting as a function of intensity of exercise. This result is consistent with the qualitative data collected by Kolnes [26] and Bratland-Sanda et al. [19] which found that patients with AN tended to under-report their light PA in discussion with the researchers. However, it is notable that this finding contrasts with data showing that non-clinical samples are more accurate when reporting lighter intensity PA [18]. One possible explanation could be that patients with AN hold a different definition of ‘light activity’ to other groups. However, this hypothesis warrants further investigation. Future studies could reduce the potential for differential definitions to affect data by providing detailed instructions and examples to help clarify what kinds of activity would be categorised as ‘light’ in intensity.

Results indicated a positive association between higher levels of compulsive exercise (CET score) and higher accuracy on overall PA. This finding is consistent with previous research, which suggests that patients who engage in exercise of this quality are more likely to be rigid in their exercise regime and more likely to keep records [24, 26]. It may be the case that patients with compulsive exercise are more self-aware of their participation in PA and thus are better able to accurately report this on self-report measures.

Motivation to change (ANSOCQ stage of change score) was not related to accuracy on the self-report measure of total PA. However, there was a weak significant relationship between increased accuracy and lower motivation to change, and this effect was particularly strong for moderate intensity PA. This is inconsistent with previous research by Kolnes [26] and Bratland-Sanda et al. [19] who both hypothesised that participants may be more inaccurate in their self-report when they are less motivated to change and comply with treatment. Further, the finding that patients who were more motivated to change were more likely to under-report moderate intensity PA is counter-intuitive, and it is not clear why this effect would be limited to only moderate intensity exercise. One factor which may explain this finding is the limited variation in the data- all patients in the sample rated themselves as being between the contemplation and action stage of change. However, no participants were on the extreme ends of the scale (pre-contemplation and maintenance stage). This lack of variation may have meant that any effect of motivation to change was not captured. Another possible factor that could influence motivation to change is level of ED symptomatology, however in this study we did not find a significant association between ED symptomatology and accuracy of reporting. Future research could replicate these analyses with a sample of patients in different stages of motivation to change to investigate this further.

Level of psychological distress (anxiety and mood symptoms on the K^− 10^) was not found to be related to under-reporting of total PA on self-report measures (i.e. the level of under-reporting was similar for those with high and low levels of distress). Interestingly, there was a slight, but non-significant trend towards higher distress being associated with more accurate reporting. Results indicated that this trend was driven by a significant relationship between higher distress and more accurate reporting of light PA. This result was inconsistent with the literature reporting that depression and anxiety symptoms may be associated with cognitive deficits in memory, which would be expected to lead to inaccurate reporting. Furthermore, it is not clear why the correlation between increased accuracy and increased distress was only significant for light intensity PA. One possible explanation for this could be that those who accurately reported their light PA had higher levels of self-focused attention and thus were more aware of the quantity of light PA they were engaging in, and may not deem it to be ‘good enough’ as it was not high intensity. It could be the case that this judgement could result in higher levels of psychological distress if the aim of exercise was to expend as much energy as possible. Self-focused attention has been associated with negative affect [49], which provides further support for this idea. However, this finding warrants further investigation.

The current study had several strengths. It was novel as it was the first study to evaluate the EPSQ compared to an objective measure of PA in a sample of outpatients with AN. It was also the first study to measure the association between clinical characteristics and inaccurate reporting of PA. Another strength of the study was the use of accelerometer devices as an objective measure of PA. Accelerometer devices are considered the most reliable measure of PA to be used in a clinical setting for this population [15]. Furthermore, participants adhered to the study protocol – average wear time for the accelerometer was 14.4 h a day for an average of 3.5 days. Given PA was not a primary outcome of the larger RCT, this was a relatively long wear-time. Wear time was similar to other studies in the area, which have ranged from 3 days (16) to 7 days [19–21]. Furthermore, Trost, McIver, and Pate [50] reported that 3–5 consecutive days of wear time reliably estimated habitual PA. However, it is recognised that while accelerometer devices are relatively practical and valid in comparison to other direct or objective measures, accelerometry is also reliant on the patient’s compliance with wearing the device [15]. Future research could consider applying stricter thresholds for time and days wearing the device to increase the accuracy of data. Furthermore, it may be more reliable to use a waterproof accelerometer device in future studies so that water-based activities, such as swimming, could be recorded.

The present research has several important clinical implications in the assessment and treatment of patients with AN. Firstly, the data suggest that clinicians should be aware that patients with AN may vary in the accuracy of their self-reported PA depending on the intensity of exercise (i.e. light, moderate, or vigorous), such that they may be more likely to under-report light PA compared to moderate or vigorous PA.

Secondly, there is preliminary evidence to suggest that the EPSQ may be an accurate measure of patients’ moderate and vigorous PA. This is clinically relevant as the EPSQ appears to be an easy to administer, efficient, and inexpensive tool that can be used in a clinical context. However, the EPSQ may not be useful in determining overall PA due to the patients’ tendency to under-report light PA on this measure, leading to a significant under-reporting of overall PA. Given the significant under-reporting of light PA on the EPSQ, it may be necessary to use another measure for capturing PA, particularly if the clinician suspects the patient may be engaging in excessive light PA. Results from the study demonstrated that accelerometers can more accurately measure light PA, although they can be time and cost-intensive to administer and interpret, as well as potentially intrusive for patients [18]. =Alternatively, a self-report measure which is more sensitive to light PA could be developed as part of future research.

Thirdly, findings suggest that patients whose exercise could be considered compulsive in quality, as measured by the CET scores, are more likely to accurately report their exercise than those whose exercise is less compulsive. Given the time and economic resources required when using accelerometer devices, it may be more important to prioritise their use to monitor patients whose PA is not classified as compulsive. Finally, the results provide preliminary evidence that clinicians should not assume that clinical characteristics such as psychological distress and motivation to change affect the accuracy of self-report PA.

The limitations of the current data are first that self-report PA data involved asking participants to reflect on their exercise over the previous month, then averaged to calculate a daily exercise average. As such, it relied heavily on participants’ recall and was not administered in respondent based interview format as in its original use. This could in part explain the significant under-reporting of light PA (as opposed to moderate and vigorous PA) as it could be argued that light PA may be more easily forgotten as it is less strenuous and possibly more likely to be incidental. Further, the days represented in the patients’ self-report were not the same days that the accelerometer was worn, and self-reported data reflected patient’s recall of PA over the previous month while the accelerometer data was over an average of four days, meaning the length of time represented also differed. Researchers asked participants to complete a written diary style record of their activity whilst wearing the accelerometer, however there was not an adequate amount of written data suitable for inclusion in the study. Future studies could consider administering a modified version of the EPSQ measure daily, and asking the participant to reflect the same days that the accelerometer device was worn. This could also assist in future studies as a way of measuring participant’s perception of how reflective the accelerometer data would be of their self-reported PA. Further, the sample was limited as it included 34 participants (with only two males) and analyses were only conducted on patients who agreed to wear an accelerometer device and complied with wear-time instructions. It is likely that this is a unique sample, and that those who did not agree to wear an accelerometer device could differ in the accuracy of their self-reported PA. An important consideration is that there were no consequences of patients reporting high or low PA, or engaging in more or less PA than usual. This is because only the researchers reviewed the accelerometer and self-report data, and data was collected at baseline limiting treatment interference. However, we do acknowledge there may be a small motivation/compliance factor, as therapists collected the accelerometers and questionnaires from the patients. It is not clear how the current data would generalise to situations where the patient’s clinician reviewed the self-report data, or if there were prescribed consequences for increased PA (e.g. increased caloric intake) in a therapy context. Future research could therefore consider testing the accuracy of the EPSQ in different settings (i.e. inpatient vs outpatient) and with patients at different stages of illness. Finally, given the small sample size included in the current study, there are limitations to the conclusions which can be drawn from the correlational analyses presented. Future studies could consider repeating the analysis with a larger sample to increase the strength of conclusions which can be drawn from correlational trends.

## Conclusion

The present study has furthered our understanding of the relationship between self-reported PA and accelerometer data in outpatients with AN. Overall, results confirm previous research findings that patients with AN underestimate their PA on self-report measures. Results also showed that patients accurately self-reported moderate and vigorous PA, but significantly under-reported light PA on the EPSQ. Furthermore, higher levels of compulsive exercise were significantly associated with more accurate reporting of total PA. Clinical features, such as motivation to change and distress were less clearly associated with the accuracy of total self-report PA, but results suggest they may be associated with inaccurate reporting at different levels of intensity of PA. Associations between clinical features and the accuracy of self-report in the current study were weak and based on a relatively small sample size, and therefore further investigation is warranted before strong conclusions can be drawn. The results have clinical implications in understanding the utility, and potential shortcomings, of self-reported PA measures, and when accelerometer devices may be more appropriate. Results may also guide future research in understanding why patients under-report PA, which could help inform the development of self-report measures and treatment programs for patients with AN.

## Data Availability

Data are available from Phillipa Hay for collaborative projects and as auspiced by the Western Sydney University Human Research Ethics Committee.

## Abbreviations

AN: Anorexia Nervosa
ANSOCQ: Anorexia Nervosa Stages of Change Questionnaire
BMI: Body Mass Index
CET: Compulsive Exercise Test
EDE: Eating Disorder Examination
EPSQ: Exercise Participation Screening Questionnaire
IPAQ: International Physical Activity Questionnaire
LEAP: CompuLsive Exercise Activity TheraPy Programme.
PA: Physical Activity
RCT: Randomised Controlled Trial
